# Mutational landscape of multiple primary lung cancers and its correlation with non-intrinsic risk factors

**DOI:** 10.1038/s41598-021-83609-y

**Published:** 2021-03-11

**Authors:** Motohiro Izumi, Jun Oyanagi, Kenji Sawa, Mitsuru Fukui, Koichi Ogawa, Yoshiya Matsumoto, Yoko Tani, Tomohiro Suzumura, Tetsuya Watanabe, Hiroyasu Kaneda, Shigeki Mitsuoka, Kazuhisa Asai, Masahiko Ohsawa, Nobuyuki Yamamoto, Yasuhiro Koh, Tomoya Kawaguchi

**Affiliations:** 1 Department of Respiratory Medicine, Graduate School of Medicine, Osaka City University, 1-4-3 Asahi-cho, Abeno-ku, Osaka, 545-8585 Japan; 2 Internal Medicine III, Wakayama Medical University, 811-1 Kimiidera, Wakayama, Wakayama 641-8509 Japan; 3 Laboratory of Statistics, Graduate School of Medicine, Osaka City University, Osaka, Japan; 4 Department of Clinical Oncology, Graduate School of Medicine, Osaka City University, Osaka, Japan; 5 Department of Pathology, Graduate School of Medicine, Osaka City University, Osaka, Japan

**Keywords:** Non-small-cell lung cancer, Oncogenes

## Abstract

Multiple primary lung cancers (MPLCs) harbour various genetic profiles among the tumours, even from individuals with same non-intrinsic risk factors. Paired mutational analyses were performed to obtain a census of mutational events in MPLC and assess their relationship with non-intrinsic risk factors. Thirty-eight surgical specimens from 17 patients diagnosed as MPLC were used. Extracted DNAs were sequenced for somatic mutations in 409 cancer-associated genes from a comprehensive cancer panel. We statistically analysed the correlation between each driver mutation frequency and non-intrinsic risk factors using Fisher's exact test, and whether genetic mutations occurred concomitantly or randomly in MPLC using an exact test. Comprehensive genetic analyses suggested different mutation profiles in tumours within the same individuals, with some exceptions. *EGFR*, *KRAS, TP53*, or *PARP1* mutations were concomitantly detected in some MPLC cases. *EGFR* mutations were significantly more frequent in never or light smokers and females. Concomitant *EGFR* or *KRAS* mutations in MPLCs were significantly more frequent than expected by chance (*P* = .0023 and .0049, respectively) suggesting a more prominent role of non-intrinsic risk factors in *EGFR* and *KRAS* mutations than other mutations, which occurred more randomly. Concomitant *EGFR* or *KRAS* mutations were particularly prominent in never or light smokers and males.

## Introduction

Lung cancer is the leading cause of cancer-related death worldwide^1^. Cancer risk factors include intrinsic and non-intrinsic factors. The majority of cancer risk factors (60–90%) are non-intrinsic^2,3^. Intrinsic risk has been defined as intrinsic DNA replication errors which are unmodifiable and occur in the process of normal human cells division. Non-intrinsic risk is defined as factors that consist of modifiable exogenous factors such as lifestyle, radiation, chemical carcinogens, tumour causing viruses, and partially modifiable endogenous factors such as biological aging, inflammation, immune responses, hormones, and metabolisms. Epidemiological studies of lung cancer have revealed multiple risk factors that comprise a combination of genetic and external factors (environmental and occupational). In particular, smoking is the main cause of the development and progression of lung cancer^4,5^.

The development of a variety of reliable and powerful molecular tools has led to the discovery of driver mutations associated with the development of lung cancer and revealed differences in the frequency of genetic mutations due to non-intrinsic factors. For example, epidermal growth factor receptor (*EGFR*) mutations are more frequently found in female patients who are never smokers and who have adenocarcinoma histology, whereas *KRAS* mutations are more common in adenocarcinoma patients who are smokers^6–8^. Furthermore, fusions of canonical oncogenes are reportedly often acquired in the early decades of life^9^. The authors suggested that these events likely take place in normal cells with competent DNA damage response^9^. Another study suggested that the majority of cancer risk is due to bad luck, with random mutations arising during DNA replication in normal, noncancerous stem cells^10^. These previous studies analysed patients with different molecular and clinical backgrounds, and so were hindered by the problem that various factors are intricately intertwined. Therefore, we focused on multiple primary lung cancers (MPLCs), which occur in a patient exposed to the identical risk factors and systemic reactions, including the immune response. This focus allowed us to elucidate the relationships between genetic mutations and non-intrinsic factors.

It is reported that MPLCs occur in 0.2 to 20% of all primary lung cancer cases, and the incidence rate has risen because of the incorporation of high-resolution computed tomography (CT) and positron emission tomography/CT (PET/CT) into clinical practice^11,12^. The initial criteria published in 1975^13^ defined MPLCs based on histology and tumour locations. However, in some cases, it is difficult to differentiate MPLCs from metastases in accordance with these criteria. In particular, bronchioloalveolar carcinomas appearing as multiple pure ground-glass opacity lesions are commonly defined as MPLC without pathological confirmation for every lesion^14^. Therefore, differentiation of MPLC from intrapulmonary metastasis is often a problem. Array comparative genomic hybridisation (CGH), histological subtyping, and imaging features are powerful tools to differentiate MPLC from intrapulmonary metastasis^15–17^.

Although next generation sequencing (NGS) enables comprehensive gene mutation analysis, in MPLC the difference of mutation profiling among multiple lesions is unclear. Therefore, we assumed that genetic mutations that are strongly influenced by non-intrinsic risk factors would occur concomitantly in multiple tumours within the same individuals, whereas the mutations that are not influenced by non-intrinsic risk factors would occur randomly.

In this study, we performed comprehensive mutational analyses for MPLC patients to clarify whether genetic mutations can occur concomitantly or randomly in multiple tumours within the same individuals. In addition, we researched if non-intrinsic factors, mainly smoking status, obesity, age, and sex, can change the occurrence of mutations.

## Methods

### Patients and sample preparation

This study involved 34 patients who underwent surgery at Osaka City University Hospital for early stage non-small cell lung cancer (NSCLC) between October 2007 and March 2019. The patients were diagnosed with MPLC based on the criteria mentioned in previous studies^13,18^. Multiple tumours in the same lobe were included only if the tumours were located within different segments, classified as origin from carcinoma in situ, exhibited different histologic features (for example, adenocarcinoma and squamous cell carcinoma), or demonstrated the same histologic features but different subtyping (for example, acinar and papillary growth patterns). All available information was carefully reviewed and considered, including radiological and pathological findings from a multidisciplinary tumour board, which included radiologists, thoracic surgeons, and medical oncologists. Pathological staging was performed using the eighth edition of the TNM Classification of Malignant Tumours. The patients provided written informed consent for the genetic research studies, which were performed in accordance with protocols approved by the Institutional Review Board at Osaka City University Hospital and Wakayama Medical University Hospital. The specimens were reviewed to ensure tissue adequacy (> 10% tumour nuclei) before testing. DNA was extracted from unstrained formalin-fixed paraffin-embedded (FFPE) resections using the QIAamp DNA FFPE Tissue Kit following the manufacturer’s instructions (Qiagen). Genomic DNA concentration was measured using a Qubit fluorometer (Thermo Fisher Scientific). We checked the degree of DNA decomposition using TapeStation (Agilent) and excluded samples that were clearly undergoing DNA degradation. Finally, we analysed 38 surgical specimens from 17 patients (see Supplementary Fig. S1 online).

### Targeted sequencing and data analysis

Next-generation sequencing for the detection of actionable somatic mutations was carried out as previously described^19^. Briefly, Ion AmpliSeq Library Kit Plus (Thermo Fisher Scientific) was used for library construction followed by barcode ligation using the Ion Xpress Barcode Adapters Kit (Thermo Fisher Scientific). Then, library samples were purified (Agencourt AMPure XP reagent, Beckman Coulter) and quantified (Ion Library Quantitation Kit, Thermo Fisher Scientific). The libraries were templated using Ion 540 Kit-Chef (Thermo Fisher Scientific) and sequencing was carried out on the Ion GeneStudio S5 (Thermo Fisher Scientific) for somatic mutations in 409 cancer-associated genes (Ion AmpliSeq Comprehensive Cancer Panel) (see Supplementary Fig. S2 online). Data analysis was conducted using the Ion Reporter Server System (Thermo Fisher Scientific) and CLC Genomics Workbench version 9 (CLC bio, Aarhus, Denmark). Visual inspection was performed to confirm the sequence data using the Integrative Genomics Viewer.

Single Nucleotide Polymorphisms (SNPs) registered in the COSMIC database^20^ or Japanese Multi Omics Reference Panel^21^ were excluded. Nonsynonymous variants with coverage of < 250 and allele frequency (AF) < 3% were excluded. And then considering about base substitution by the FFPE sample, regarding C > T/G > A base substitution, those with AF of less than 5% and those with no reported lung cancer in the COSMIC and TCGA databases were excluded.

### *ALK* immunohistochemistry (IHC)

Patients lacking *EGFR* and *KRAS* mutations were examined for *ALK* fusions. FFPE specimens were used for IHC with the Histofine ALK iAEP kit (Nichirei Bioscience, Tokyo, Japan). *ALK* IHC results were classified into positive (positive tumour cells > 0%) and negative (0%).

### *ROS1* fusion gene detection

Patients lacking *EGFR* and *KRAS* mutations were examined for *ROS1* fusions. Using haematoxylin and eosin-stained tissue slides as a guide, the corresponding areas of tumours on six sections of 5-μm-thick FFPE specimens were marked and scraped off the slide for macrodissection. Real-time reverse transcription-polymerase chain reaction was performed using RNA extracted from macrodissected specimens at LSI Medience (Osaka, Japan).

### Array CGH

In cases where NGS showed the same mutation profiling or the same driver mutations (*EGFR* or *KRAS* mutations, or *ALK* or *ROS1* rearrangements), array CGH was performed at DNA Chip Research Inc. (Tokyo, Japan). FFPE DNA samples (20 ng) were amplified using the GenomePlex Complete Whole Genome Amplification Kit (WGA2; Sigma-Aldrich, St. Louis MO, USA), according to the manufacturer's instructions. Each amplified sample (250 ng) was labelled with SureTag Complete DNA Labelling Kit (Agilent Technologies). In brief, Cy3- and Cy5-labelled DNA were combined with Cot-1 DNA (Thermo Fisher Scientific) and CGH blocking agent (Agilent Technologies), and then denatured and hybridised to the arrays (SurePrint G3 Human CGH Microarray 8 × 60 K; Agilent Technologies) for 24 h in a rotating oven at 67 °C and 20 rpm (Agilent Technologies). After hybridisation and washing, the microarray was scanned using a model G4900DA SureScan Microarray Scanner System (Agilent Technologies). Images were analysed with Feature Extraction Software 12.1.1.1 (Agilent Technologies) with the CGH_1201_Sep17 protocol for background subtraction and normalisation. Data analysis of the microarray experiments was conducted using the Aberration Detection Method-2 statistical algorithm (Agilent Technologies) on the basis of the combined log2 ratios at a threshold of 6.0, as was done in a previous study^22^. The data were centralised and calls with average log2 ratios of < 0.3219 were filtered to exclude false positives.

### Statistical analysis

The correlation between the frequency of tumours carrying each driver mutation and non-intrinsic risk factor was analysed using Fisher’s exact test. Statistical analyses were performed to determine whether genetic mutations would occur concomitantly or randomly in multiple tumours using an exact test. At first, we assumed genetic mutations occurred by chance and calculated the frequency of each mutation in both, either, or neither lesions based on the Japan Molecular Epidemiology for lung cancer (JME) study, which was a prospective and multicentre molecular epidemiology study for Japanese NSCLC patients^7^. The frequency of mutations not analysed in the JME study was calculated from the number of samples in this study. Supplementary Table S1 online shows the frequency of each mutation. The assumed frequency of mutations was compared with the actual data to statistically analyse the difference in how the mutations occurred. If there was a significant difference, the mutations would occur concomitantly in the multiple tumours within the same individuals. Cases that had more than three lesions were considered discrepant if all lesions did not share the same mutation for purposes of this analysis. Statistical significance was assumed for a two-tailed p-value < 0.05.

## Results

### Patient characteristics

We obtained 78 surgical specimens from 34 patients who were pathologically diagnosed as MPLC. Among them, 38 specimens from 17 patients were eligible for sequencing (see Supplementary Fig. S1 online). In addition to the clinicopathological features, patients’ age, sex, smoking history, body mass index (BMI), radiologic features, and surgical procedures were reviewed. We defined light smokers as Brinkman Index (BI) < 200, medium smokers as BI 200 to 600, and heavy smokers as BI > 600 in accordance with a previous report^7^. Seventeen patients were divided into groups by the following characteristics (Table 1): 12 males, 5 females; 11 ever smokers, 6 never smokers. Age ranged between 50 and 83 years (mean age 73 years). Data concerning tumour location, pathological stage, maximum diameter of the tumours, histology, and operative procedure are presented in Table 2.

**Table 1:** Patient characteristics.

Characteristic	Cases (n = 17)
Age, median (range)	73 (50–83)
**Sex, n (%)**	
Male	12 (70.6)
Female	5 (29.4)
**Smoking history, n (%)**	
Never	6 (35.3)
Ever	11 (64.7)
**Brinkman index (BI), n (%)**	
Never smoker	6 (35.3)
0 < BI < 200	1 (5.9)
200 ≤ BI < 600	1 (5.9)
600 ≤ BI	9 (52.9)
**COPD, n (%)**	
Positive	5 (29.4)
Negative	12 (70.6)
**Body mass index (BMI), n (%)**	
BMI < 18.5	3 (17.6)
18.5 ≤ BMI < 25	14 (82.4)
25 ≤ BMI	0 (0)

**Table 2:** Tumour location, pathological stage, maximum diameter of the tumours, histology and operative procedure in each patient.

Patients no.	Age	Sex	BI		Size (mm)	p-stage	Tumour location	Operative procedure	Histology (%)
1	72	f	750	A	18	IA	Rt S6	Rt S6 division segmentectomy	Lepidic (95), papillary (5)
				B	7	IA	Lt S9	Lt bottom segmentectomy	Papillary (50), acinar (40), lepidic (10)
				C	10	IA	Lt S10	Lt bottom segmentectomy	Papillary (60), acinar (30), lepidic (10)
2	83	m	1380	A	28	IA	Lt S10	Lt lower lobectomy	Papillary (70), lepidic (15), acinar (15)
				B	18	IA	Lt S10	Lt lower lobectomy	Papillary (60), acinar (40)
3	80	m	300	A	27	IB	Lt S1 + 2	Lt upper segmentectomy	Acinar (90), lepidic (5), papillary (5)
				B	22	IB	Lt S6	Lt S6 division segmentectomy	Acinar (50), lepidic (50)
4	78	m	1280	A	13	IA	Lt S9	Lt lower partial resection	Micropapillary (60), papillary (20), lepidic (20)
				B	26	IA	Rt S1	Rt upper lobectomy	Lepidic (90) acinar (10)
5	72	m	1060	A	68	IIA	Lt S6	Lt lower lobectomy	Papillary (80), lepidic (20)
				B	18	IA	Lt S3	Lt S3 division segmentectomy	Papillary (95), lepidic (5)
6	75	m	900	A	22	IA	Rt S9	Rt lower lobectomy	Adenosquamous
				B	11	IVA	Lt S1 + 2	Lt S1 + 2 division segmentectomy	Acinar (95), solid (5), STAS ( +)
7	64	m	1080	A	16	IA	Lt S9	Lt lower lobectomy	Papillary (90), lepidic (10), STAS ( +)
				B	40	IB	Rt S1	Rt upper lobectomy	Lepidic (80), papillary (15), acinar (5)
8	67	m	940	A	29	IA	Lt S3	Lt upper lobectomy	Lepidic (90), acinar (10)
				B	15	IA	Lt S6	Lt S6 division segmentectomy	Micropapillary (80), lepidic (10), acinar (10)
				C	32	IB	Rt S2	Rt upper partial lobectomy	solid (95), lepidic (5)
				D	11	IA	Rt S6	Rt S6 division segmentectomy	Papillary (60), lepidic (40)
9	68	m	2000	A	9	0	Lt S1 + 2	Lt upper partial resection	Sq
				B	15	IA	Lt S9	Lt lower partial resection	Sq
10	62	m	840	A	35	IB	Rt S8	Rt lower lobectomy	Sq
				B	18	IA	Rt S1	Rt upper partial resection	Sq
11	83	f	0	A	26	IB	Rt S3	Rt upper lobectomy	Lepidic (90), acinar (10)
				B	14	IA	Lt S1 + 2	Lt upper partial segmentectomy	MIA
12	74	f	0	A	35	IB	Rt S1	Rt S1 division segmentectomy	Acinar (80), papillary (10), lepidic (10)
				B	54	IIA	Lt S3	Lt upper lobectomy	Papillary (60), lepidic (20), Sq (20)
13	64	f	0	A	22	IA	Rt S2	Rt upper lobectomy	Lepidic (90), papillary (10)
				B	19	IA	Rt S3	Rt upper lobectomy	Lepidic (85), acinar (10), papillary (5)
14	60	f	0	A	15	IB	Lt S1 + 2	Lt upper lobectomy	Papillary (60), solid (10), acinar (15), lepidic (15)
				B	5	IB	Lt S10	Lt lower partial lobectomy	Papillary (50), acinar (40), lepidic (10)
15	72	m	150	A	22	IA	Rt S9	Rt bottom segmentectomy	Papillary (70), lepidic (20), acinar (10)
				B	18	IA	Rt S8	Rt bottom segmentectomy	AIS
				C	17	IA	Rt S10	Rt bottom segmentectomy	AIS
16	73	m	0	A	15	IA	Rt S6	Rt lower partial resection	AIS
				B	16	IA	Lt S8	Lt bottom segmentectomy	Papillary (very small amount of lepidic and acinar)
17	50	m	0	A	13	IA	Rt S10	Rt lung bottom segmentectomy	Papillary (50), lepidic (40), acinar (10)
				B	36	IB	Lt S10	Lt lung bottom segmentectomy	Lepidic (60), acinar (30), papillary (10)

*BI* Brinkman index, *f* female, *m* male, *STAS* spread through alveolar spaces, *Sq* squamous cell carcinoma, *MIA* microinvasive adenocarcinoma, *AIS* adenocarcinoma in situ.

### Targeted sequencing identifies somatic mutations in lung cancers

**Figure 1:**
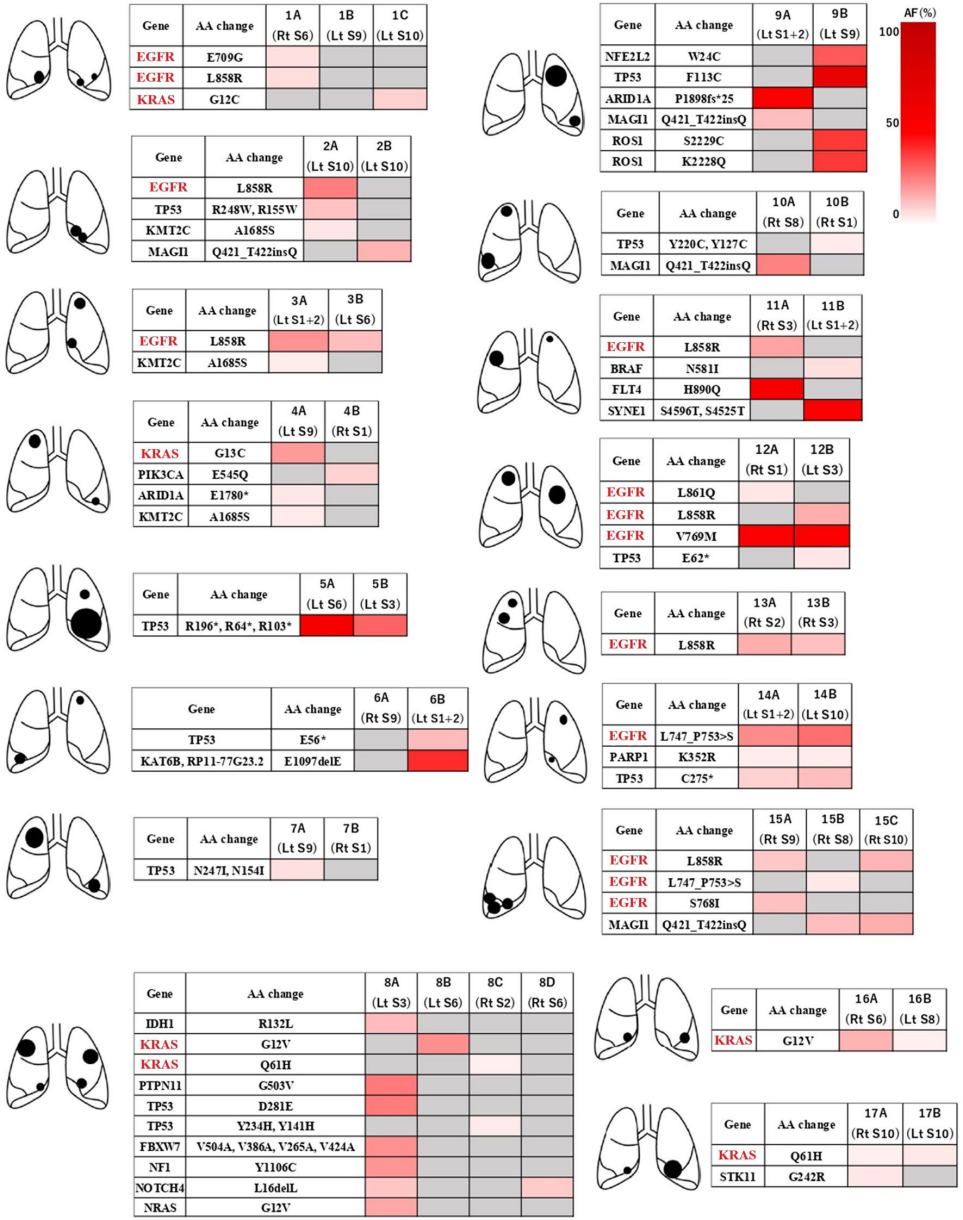
Paired mutation profiling in each patient. The gene mutations and amino acid substitutions within the individual tumours constituting the multiple lung cancers in each patient. Different mutation profiles were evident, except in four cases. The four cases showing the same mutation profiling were consistent with multiple lung cancer from the clinical data. AF, allele frequency; AA change, amino acid change**.** All figures were created using Paint 3D version 6.2009.30067.0 (https://www.softpedia.com/get/Multimedia/Graphic/Graphic-Editors/Paint-3D.shtml), and those figures were edited to 300 dpi resolution using GIMP version 2.10.20 (https://gimp.jp.uptodown.com/windows).

**Figure 2:**
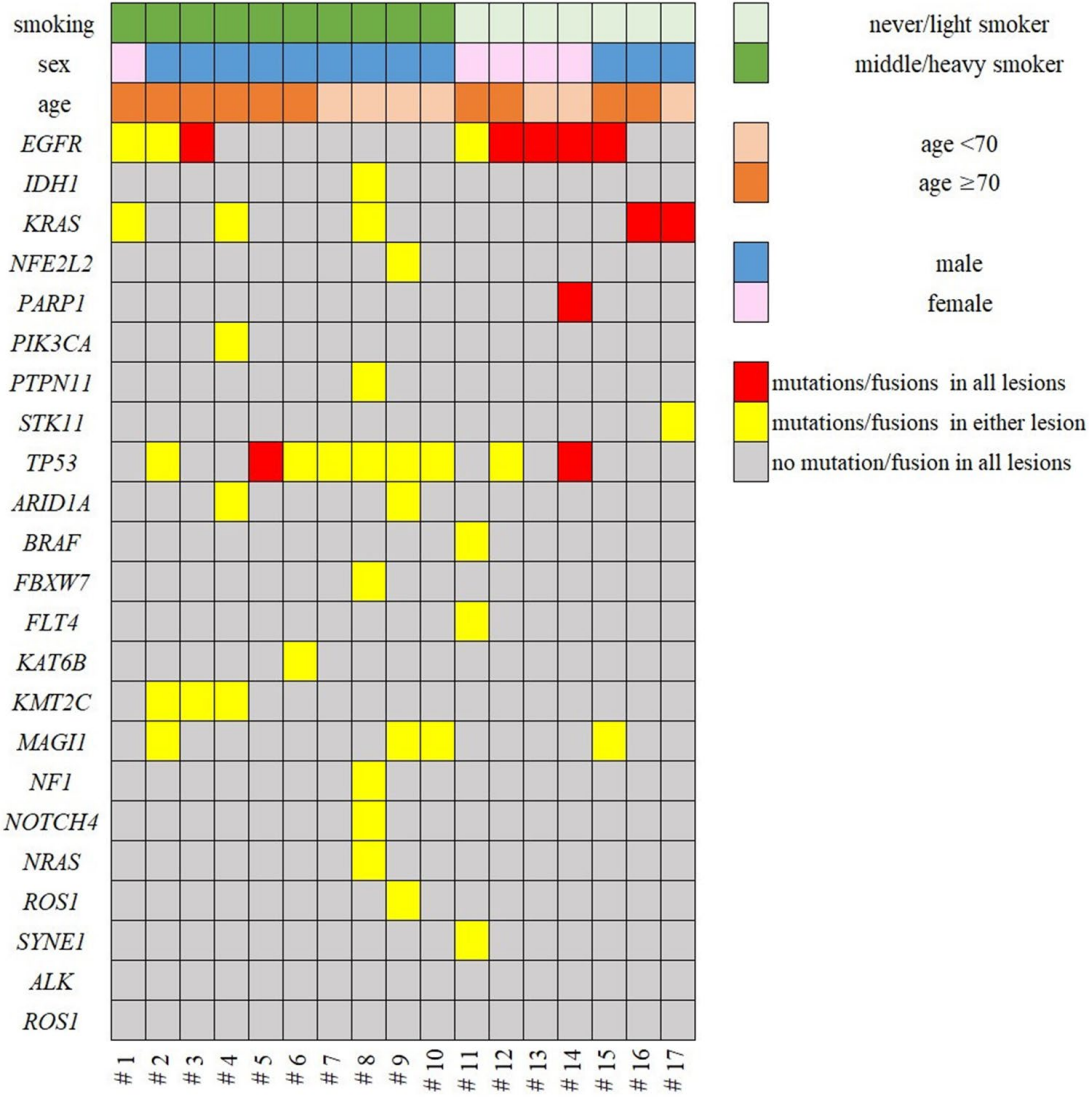
Heat map of gene mutations in 17 patients with multiple lung primary lung cancers. Samples were sequenced for somatic mutations in 409 cancer-associated genes and were analysed for *ALK* and *ROS1* fusion genes. A total of 21 mutations were detected. This map visualises the gene mutations of each patient. All figures were created using Paint 3D version 6.2009.30067.0, and those figures were edited to 300 dpi resolution using GIMP version 2.10.20.

Targeted sequencing was performed for 38 surgically resected tumours from 17 patients. Deep sequencing was successfully performed in all of the specimens. The mean coverage depth was 846-fold for tumour samples (range: 540–2061) (see Supplementary Table S2 online). In 38 specimens, sequencing analysis of 409 genes identified 21 mutations (0–7 mutations per tumour) based on the defined filter criteria (Figs. 1, 2). *ALK* and *ROS1* rearrangements were examined in 16 samples lacking *EGFR* or *KRAS* mutations. These rearrangements were not detected in any of the samples (Fig. 2).

In each patient, with a few exceptions, the gene mutations and amino acid substitutions within the individual tumours constituting the multiple lung cancers showed different mutation profiling (Fig. 1). Case 5, 13, 14, and 16 showed the same mutation profiling.

Array CGH was performed for these four cases with the same mutation profiling among paired tumours by NGS and for three cases (Cases 3, 15 and 17) with the same driver mutations that included *EGFR* or *KRAS* mutations. The results of array CGH for each of the seven cases are shown in Supplementary Fig. S3 online. Case 14 displayed a gain of chromosome 22 in one tumour. Case 16 displayed losses of chromosomes 5 and 19 in one tumour. These results would be equivocal to conclude they represented MPLCs, nonetheless, Case 14 did not share all components of pathological subtypes and their proportions and morphological features were different, and one tumour in Case 16 was AIS. Therefore, the two cases were conclusively diagnosed as MPLCs. The other cases showed the typical array CGH results, which were amplifications or deletions in one of the tumours. In Case 5, the second cancer developed more than 3 years after the first cancer, so it was consistent with metachronous MPLC. In Case 13, radiographic findings of both tumours showed ground-glass opacity with no recurrence (see Supplementary Fig. S4 online). These clinical courses also supported the diagnoses of these cases as MPLCs.

**Figure 3:**
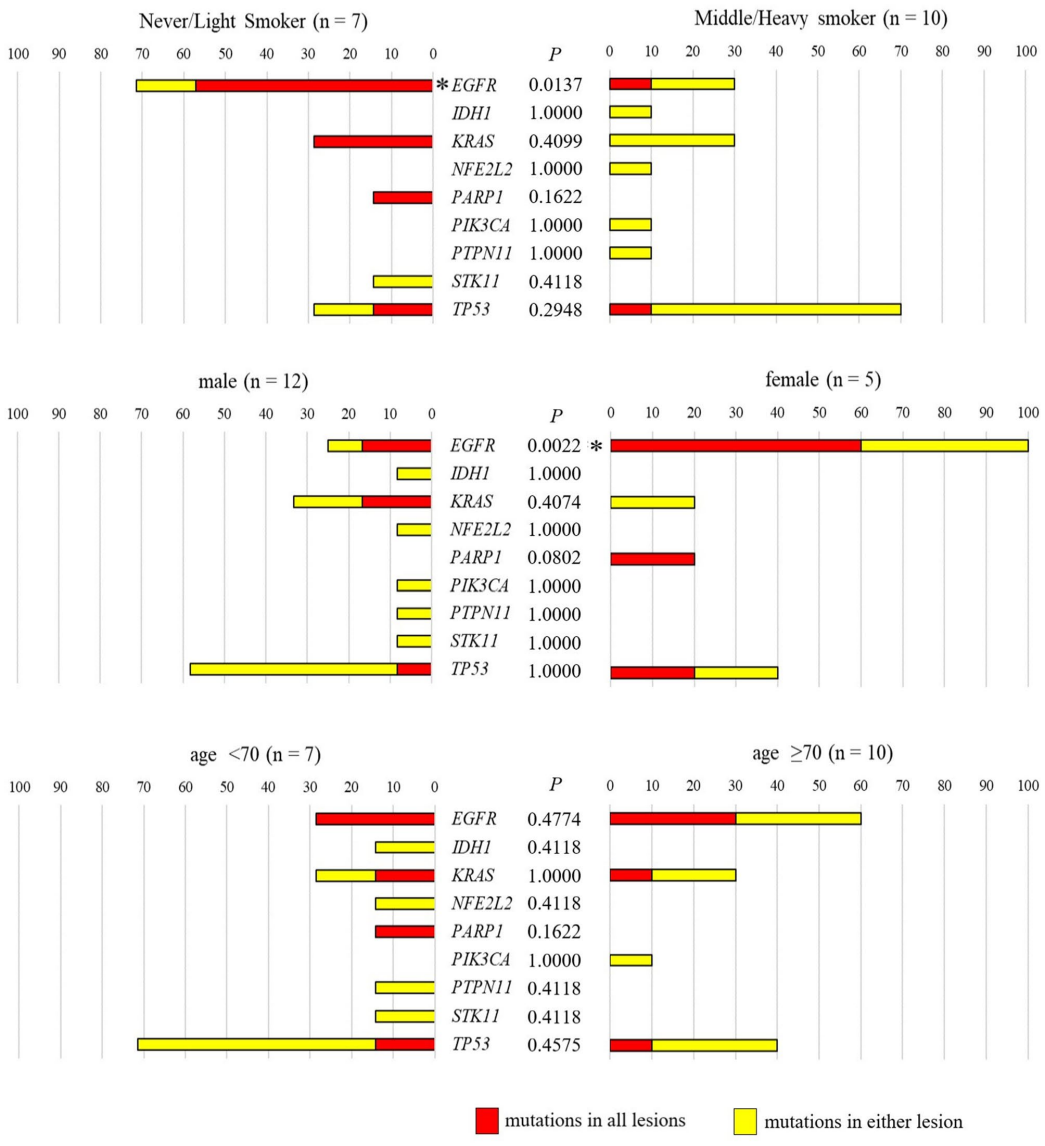
Correlation between mutations and non-intrinsic factors. In patients with MPLC, the *EGFR* mutations occurred significantly more frequent in never/light smokers and females. Asterisk (*) indicates P < .05. All figures were created using Paint 3D version 6.2009.30067.0, and those figures were edited to 600 dpi resolution using GIMP version 2.10.20.

We assessed whether non-intrinsic risk factors including age, sex, and smoking status were correlated with specific mutations. We could not assess the correlation between obesity and mutation profile, because none of the patients had a BMI > 25. In patients with MPLC, *EGFR* mutations occurred significantly more frequently in females and in never or light smokers, as well as the single primary lung cancers reported previously^7^ (Fig. 3). The other mutations had no significant correlation with non-intrinsic risk factors in patients with MPLC. Heat mapping showed that each mutation occurred in all tumours, in either tumour, or in no tumour in multiple tumours within the same individuals (Fig. 2). The *EGFR*, *KRAS*, *TP53*, and *PARP1* mutations occurred concomitantly in some cases, but the other mutations were not detected concomitantly. We set each mutation frequency of single primary lung carcinoma in each characteristic based on the JME study and the data of the present study (see Supplementary Table S1 online). We assumed the mutations occurred by chance even in MPLC. Comparing the assumed frequency of mutations with the actual data, we statistically performed paired mutational analyses using the exact test to clarify the nature of the occurrence of mutations in MPLC. The results are shown in Table 3 and Supplementary Table S3 online. *EGFR* mutations were detected in 8 of 17 patients (all lesions in 5 patients, either lesions in 3 patients, no lesion in 9 patients). The occurrence of concomitant *EGFR* mutations in multiple tumours within the same individuals was significantly more frequent than expected by chance (P = 0.0023). *KRAS* mutations were detected in 5 of 17 patients (all lesions in 2 patients, either lesions in 3 patients, no lesion in 12 patients).

**Table 3:** Number of cases in each gene mutation.

Gene	Total (n = 17)			p
	All (n)	Either (n)	Neither (n)	
*EGFR*	5	3	9	.002
*IDH1*	0	1	16	.997
*KRAS*	2	3	12	.005
*NFE2L2*	0	1	16	.702
*PARP1*	1	0	16	.009
*PIK3CA*	0	1	16	.702
*PTPN11*	0	1	16	.741
*STK11*	0	1	16	.983
*TP53*	2	7	8	.234
*ARID1A*	0	2	15	.694
*BRAF*	0	1	16	.741
*FBXW7*	0	1	16	.741
*FLT4*	0	1	16	.741
*KAT6B*	0	1	16	.741
*KMT2C*	0	3	14	.680
*MAGI1*	0	4	13	.682
*NF1*	0	1	16	.741
*NOTCH4*	0	1	16	.741
*NRAS*	0	1	16	.741
*ROS1*	0	1	16	.741
*SYNE1*	0	1	16	.741

A significant difference (P < .05) suggested the mutations would occur concomitantly, whereas no significant difference (P ≥ .05) suggested that the mutations would occur by chance in the multiple tumours within the same individuals. All, mutations in all lesions within the same individuals; either, mutations in each lesion within the same individuals; neither, no mutation in all lesions within the same individuals.

The occurrence of concomitant *KRAS* mutations in multiple tumours within the same individuals was significantly more frequent than expected by chance, although there were few cases of *KRAS* mutated lung cancers (P = 0.0049). In contrast, *TP53* mutations were detected in 9 of 17 patients (all lesions in 2 patients, either lesion in 7 patients, no lesion in 8 patients). There was no significant difference in occurrence of *TP53* mutations between the calculated frequency and the present data (P > 0.05). Therefore, *TP53* could occur randomly, even in the same individuals. Concomitant *PARP1* mutations were also significantly more frequent than expected by chance. The likely reason is that the frequency of *PARP1* mutation in lung cancer was very low and that only one patient with *PARP1* mutated lung cancer had concomitant *PARP1* mutations in MPLC. Regarding the other mutation, there was no significant difference between the calculated frequency and the present results (P > 0.05). When we analysed whether smoking status, BMI, age, and sex were concomitantly or randomly associated with occurrence of gene mutations, concomitant *EGFR* or *KRAS* mutations occurred significantly more frequently in males and never or light smokers (see Supplementary Table S3 online). Younger patients (< 70 years old) also had significantly more concomitant *EGFR* mutations than those in older patients (≥ 70 years old). Three interesting cases are detailed below.

### Case presentations

**Figure 4:**
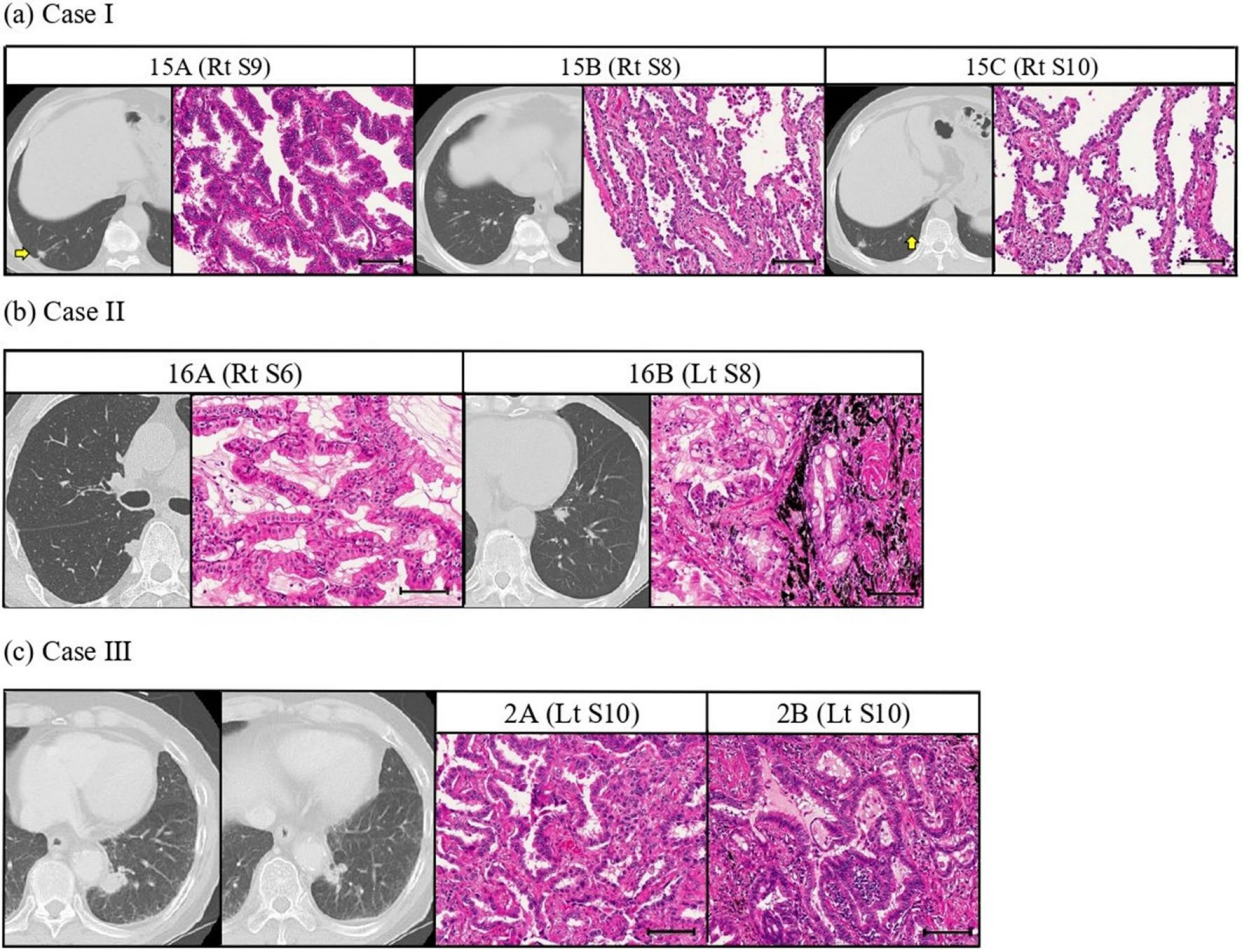
Summary of case according to radiological and pathological examination. **(a)** In case I, the tumours in right S8 and S10 were classified as adenocarcinoma in situ, and the tumour of right S9 were classified predominantly papillary adenocarcinoma. **(b)** In case II, the right S6 tumour was classified as AIS, and the left S8 tumour was classified papillary adenocarcinoma. **(c)** In case III, CT imaging showed two masses were adjacent to each other in the left S10. Both tumours were classified as predominantly papillary adenocarcinomas. However, the 2A tumour included lepidic construction and the 2B lesion contained relatively large amounts of acinar components without a lepidic component. Morphologic features were not completely similar between them. Scale bar = 100 μm. All figures were created using Paint 3D version 6.2009.30067.0, and those figures were edited to 300 dpi resolution using GIMP version 2.10.20.

Case I: A 72-year-old male who was a light smoker had triple primary adenocarcinomas in S8, S9, and S10 of the right lobe. Right basal segmentectomy was performed. The results of mutation profiling using NGS showed that the S8 tumour had *EGFR* deletion 19, whereas the S9 and S10 tumours had *EGFR* L858R. The three tumours displayed different mutation profiling patterns (Fig. 1). Pathologically, the tumours in right S8 and S10 were classified as adenocarcinoma in situ (AIS), and the right S9 tumour was classified as predominantly papillary adenocarcinoma (Fig. 4a). The patient has had no recurrence for the 12 months that have elapsed since surgery. Thus, this case was consistent with MPLC. This case suggests that some populations are prone to *EGFR* mutations, although there is the difference of amino acid substitutions.

Case II: A 73-year-old male never smoker came to our department after abnormalities in the right S6 were detected on a chest CT performed as a screening procedure before surgery for pancreatic cancer (Fig. 4b). During follow-up after resection of the right S6 lesion, multiple tumours in right S7, S9, and S10 lesions developed. Clinical distinction between the primary and metastatic tumours was difficult for the three tumours, therefore, right lower lobectomy was performed. Pathological findings showed AIS in all four tumours. Two new tumours subsequently developed in right S5 and left S8 lesions. We clinically diagnosed these tumours as MPLC and resected them. Pathologically, the right S5 tumour was classified as AIS and the left S8 tumour was classified predominantly papillary adenocarcinoma. They were also consistent with MPLC (Fig. 4b). Although tumours including abundant tumour ratio involved only two lesions (right S6 and left S8), *EGFR* mutations were clinically examined in all lesions. All displayed the *EGFR* wild type. While the possibility of the influence of passive smoke cannot be denied, this case suggests that some cases are unlikely to have *EGFR* mutation, even in never smokers.

Case III: A 83-year-old male heavy smoker came to our department after CT detected two masses adjacent to each other in the left S10 (Fig. 4c). There were no significant lymphadenopathy and metastases, so left lower lobectomy was performed. Pathologically, both tumours were classified as predominantly papillary adenocarcinomas. There were some differences between them. One tumour included lepidic construction and the other included relatively large amounts of acinar components without a lepidic component (Fig. 4c). Morphological features were also not completely similar between the two groups. Therefore, these tumours were diagnosed as double primary lung cancers. *EGFR* L858R mutation was detected in one tumour (left 10A) but not in the other tumour (left 10B) (Fig. 1). Even if *EGFR* mutations were detected in all lesions, it would be difficult to distinguish between MPLC and pulmonary metastasis. However, an *EGFR* mutation in only one lesion would support a diagnose as MPLC.

## Discussion

Our study analysing mutation profiling of MPLCs using NGS showed that concomitant *EGFR* or *KRAS* mutations in MPLCs were significantly more frequent than expected by chance, whereas the other most mutations occurred randomly. Non-intrinsic factors such as smoking status, sex, and age were considered to be factors contributing to concomitant *EGFR* or *KRAS* mutations in MPLCs.

Non-intrinsic risk factors including inherited predispositions are carcinogenic risks, and exposure to tobacco smoke is the primary etiologic factor responsible for lung cancer. However, lung cancer in never smokers comprises an estimated 15 to 20% of cases in men and over 50% in women globally^23^. The mechanisms of the occurrence of lung cancer in never smokers are unclear. Random mutations arising during DNA replication in normal, noncancerous stem cells are also considered to be carcinogenic risks^10^, although this conclusion is very controversial^2,3^. Little is known of the cause of carcinogenesis and occurrence of mutations, using multiple tumours within the same individuals and also within the same organs. MPLC is considered an appropriate model for elucidate these unknowns.

In a case with two tumours with the same matching mutations, we assume that the tumours are a consequence of metastasis because, theoretically, metastatic lesions inherit genomic characteristics^10^. Along with the development of sequencing technology, it has been suggested that genetic mutational profiling using NGS might be useful to distinguish between MPLC and intrapulmonary metastasis^24,25^. However, matched mutations may occur in double primary tumours, while additional mutations may occur in metastasis^26^. Several studies have reported intratumor heterogeneity of *EGFR* mutations^27–29^. Therefore, multiple tumours within the same patients may harbour various genetic profiling patterns, regardless of MPLC or intrapulmonary metastasis. Owing to tumour heterogeneity and insufficient understanding of their clinicopathological characteristics, there are currently no golden diagnostic criteria for MPLCs. Array CGH has been confirmed as a powerful method for the study of DNA copy number alterations in a variety of cancer types^30^. Comparing paired tumours in the somatic allelic gains and losses across the genome using array copy number data has provided evidence to classify tumour pairs as clonal metastases or as independent multiple primary tumours^15^. Additionally, comprehensive histological subtyping and morphological features, including nuclear pleomorphism, cell size, acinus formation, nucleolar size, mitotic rate, nuclear inclusions, intraalveolar clusters and necrosis, are tools to differentiate MPLC from intrapulmonary metastasis^17,31^.

We therefore checked for histologic subtypes and morphologic features in pathological findings as well as CT findings suspected of intrapulmonary metastasis, such as well circumscribed, rounded lesions, feeding vessel sign, and lymphadenopathy common in multiple lesions. We then carefully excluded cases of intrapulmonary metastasis from our analysis, regardless of the sequencing data, and confirmed the absence of paired tumours with strikingly similar morphologic features, especially in cases with similar patterns of histologic subtypes, such as Cases 1, 2, 5, 9 and 10. Furthermore, we verified that the diagnosis was consistent with MPLC using array CGH in some cases.

Presently, *EGFR* mutations significantly occurred concomitant with MPLC. This finding suggests that the existence or absence of *EGFR* mutations will not impact on diagnosis of MPLC, intrapulmonary metastasis, or recurrence tumours. One of the reasons why the mutations occurred concomitantly may be the association with germline mutations and SNPs. Previous reports have identified germline mutations in driver oncogenes that are associated with lung cancers, such as *EGFR*^32–36^ and human epidermal growth factor receptor 2 (*HER2*)^37^, which suggests that a heritable predisposition to lung cancer is a contributor in some cases. *EGFR* V769M was demonstrated to be a germline related to MPLC and that harbours co-occurring somatic mutations in *EGFR*^35^. Presently, *EGFR* V769M was detected in a patient (Case 12) and a co-occurring somatic variant in exon 21 (L861Q or L858R) was found (Fig. 1). And then, it was reported that Six loci, represented by seven SNPs (rs2736100 at 5p15.33, rs2853677 at 5p15.33, rs2179920 at 6p21.32, rs3817963 at 6p21.3, rs7636839 at 3q28, rs7216064 at 17q24.3, and rs2495239 at 6p21.1) have been associated with the risk of lung adenocarcinomas with *EGFR* mutation^38^. There were no significant differences in the association of these seven SNPs with gender or smoking status, suggesting that these loci likely affected the risk for *EGFR*-mutated lung adenocarcinomas, irrespective of gender and smoking status^38^. The sequencing panel used in this study targeted only on the somatic mutations and we were unable to examine these SNPs. However, some patients with *EGFR*-mutated lung cancer would be strongly associated with non-intrinsic factors, including SNPs. *KRAS* mutations also occurred concomitantly in MPLC in this study. Although SNPs related with *KRAS* have been reported^39^, the importance is unclear since other studies did not find such a relationship^40^. *KRAS* mutations were also presently detected in never or light smokers. This finding could reflect the small number of *KRAS* mutated cases, ambiguous information concerning smoking status because of patient’s self-reporting, and a lack of information of passive smoking history. Further validation is needed. The present finding that *TP53* mutations occurred randomly in multiple tumours within the same individuals suggests that *TP53* mutations are not commonly associated with non-intrinsic factors. There were few cases with the other mutations, which hindered evaluation. However, the present finding is consistent with the previous report of the extreme rarity of these mutations in single lung cancers. Thus, it is considered they were less affected by non-intrinsic factors, and that the rare mutations occurred by chance.

This study has several limitations. First, our sample size was relatively small, and the retrospective nature of the study might have induced a selection bias. Thus, further studies using a larger cohort is warranted to confirm the results and to reveal more detailed genetic features and complexities of MPLC. However, MPLC is a relatively rare disease, and our assessment involved unique statistical analysis and datasets of samples from patients with MPLC. Secondly, most C > T/G > A transitions were associated with a low variant allelic frequency, suggesting that this mutational pattern was an artefact related to formalin fixation. A lack of normal tissue reference made difficult to assess the SNPs, germline mutations, and somatic mutations. Therefore, we used relatively strict filtering criteria. Finally, array CGH using WGA methods potentially had an amplification bias.

Although a larger prospective study is needed to assess these results, they are important as they are the first assessment of whether genetic mutations can occur concomitantly or randomly in multiple tumours within the same individuals. Validation of the existence of concomitant mutations would be useful for accurate diagnosis, staging, and therapeutic strategy. The findings of the present MPLC study confirms that some cases with *EGFR-* or *KRAS*- mutated tumours are strongly related to non-intrinsic factors and suggests that the other most mutations may occur by chance.

## Supplementary information

Supplementary information.
