# Supplementary material for: Mutational landscape of multiple primary lung cancers and its correlation with non-intrinsic risk factors
Source: Sci Rep. 2021 Mar 11;11:5680. doi: 10.1038/s41598-021-83609-y (PMC7952588; doi:10.1038/s41598-021-83609-y)
Supplement: Supplementary file 1 — Supplementary information. [file 41598_2021_83609_MOESM1_ESM.docx]

**Mutational landscape of multiple primary lung cancers and its correlation with non-intrinsic risk factors**

**Motohiro Izumi, Jun Oyanagi, Kenji Sawa, Mitsuru Fukui, Koichi Ogawa, Yoshiya Matsumoto, Yoko Tani, Tomohiro Suzumura, Tetsuya Watanabe, Hiroyasu Kaneda, Shigeki Mitsuoka, Kazuhisa Asai, Masahiko Ohsawa, Nobuyuki Yamamoto, Yasuhiro Koh, Tomoya Kawaguchi**


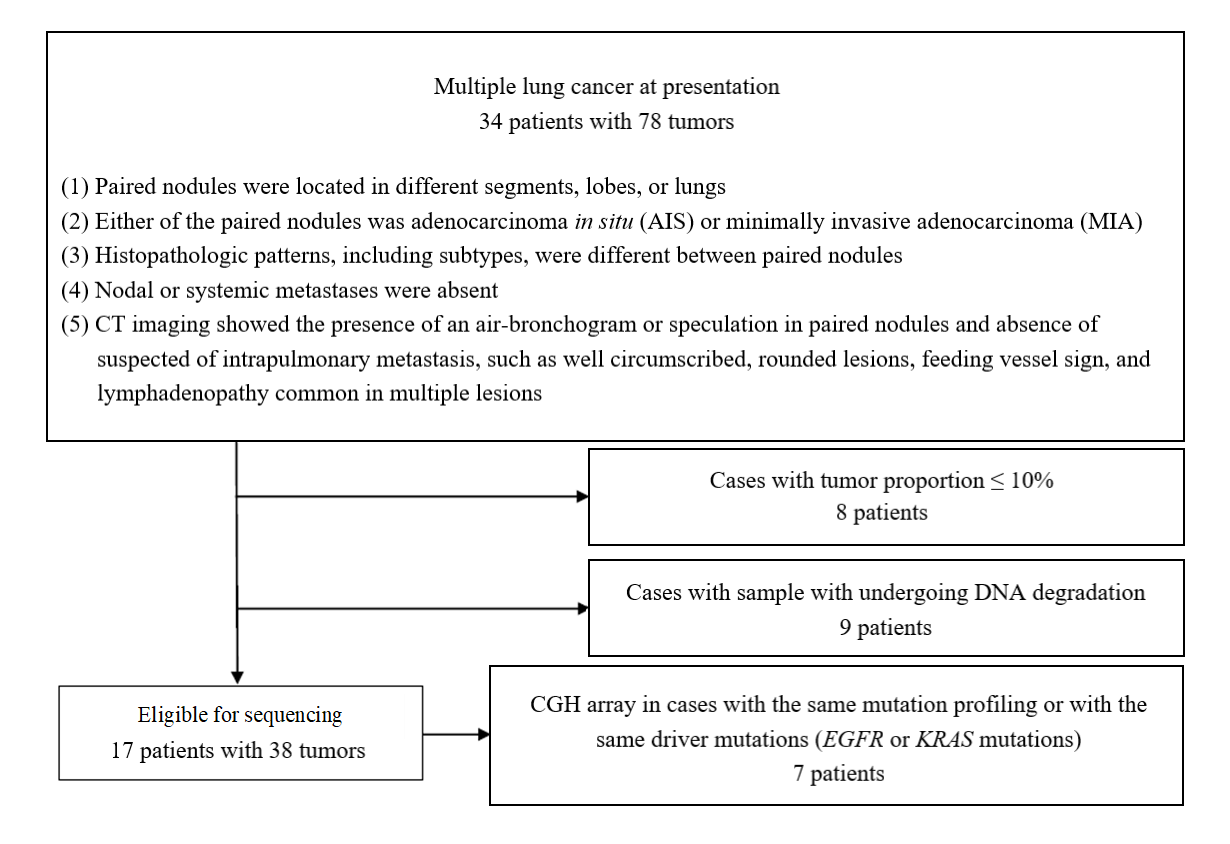


**Supplementary Fig. S1.** Approach used in studying with multiple lung cancer. Out of 34 patients with 78 tumours, samples with tumour proportion ≤10 % and apparent DNA degradation were excluded. Array comparative genomic hybridisation (CGH) was performed for 14 paired specimens from seven patients who shared the same gene mutations, including driver mutations such as *EGFR* and *KRAS*, between tumours.


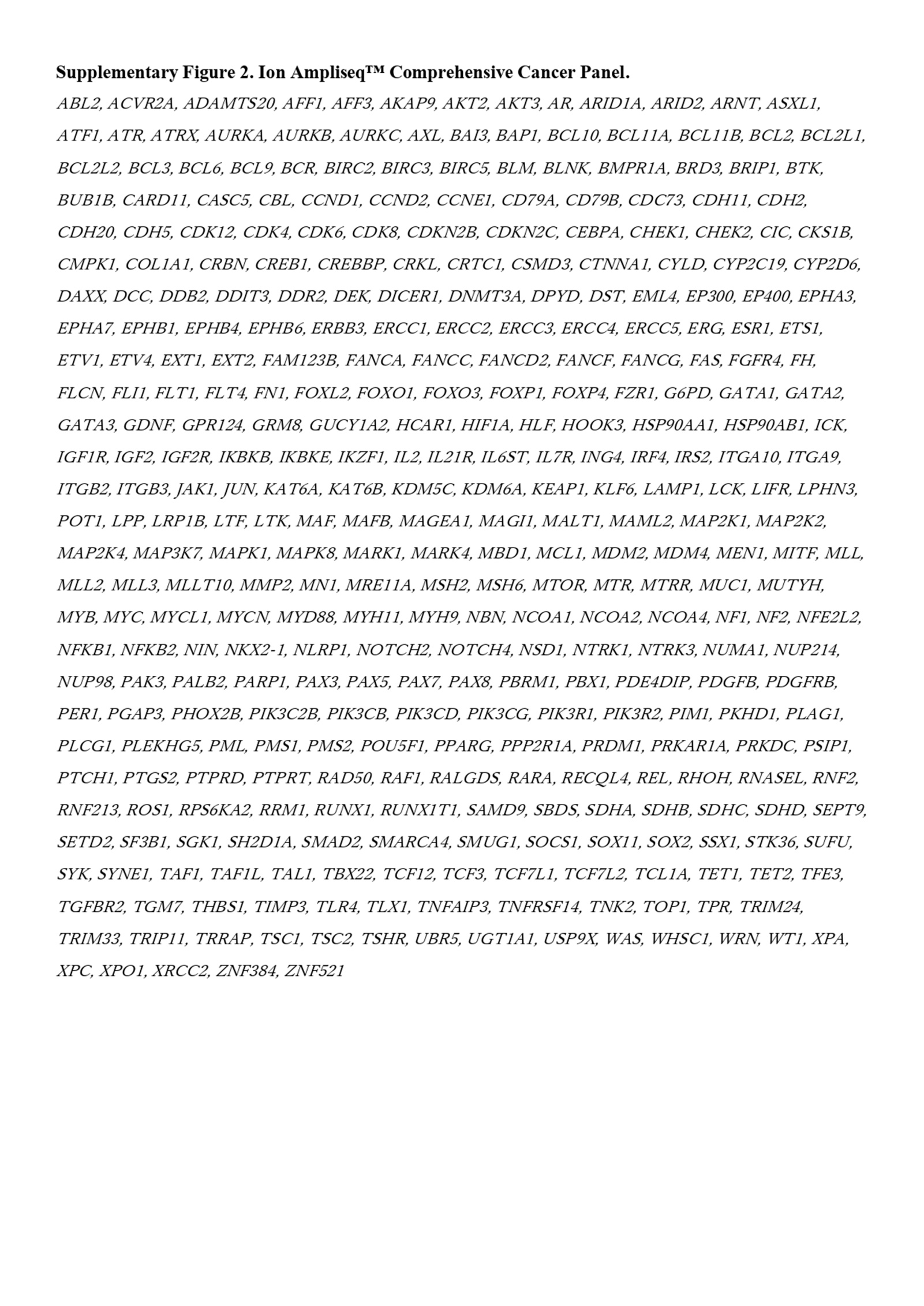


**Supplementary Fig. S2.** Ion Ampliseq Comprehensive Cancer Panel. Sequence analyses identified 409 somatic mutations.

Case 3


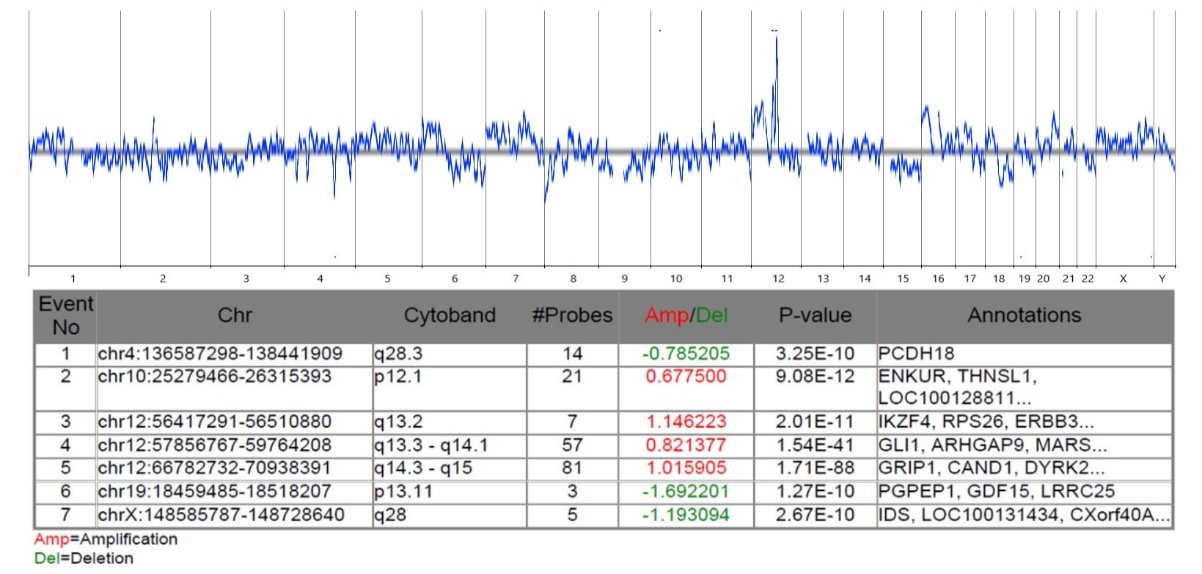


Case 5


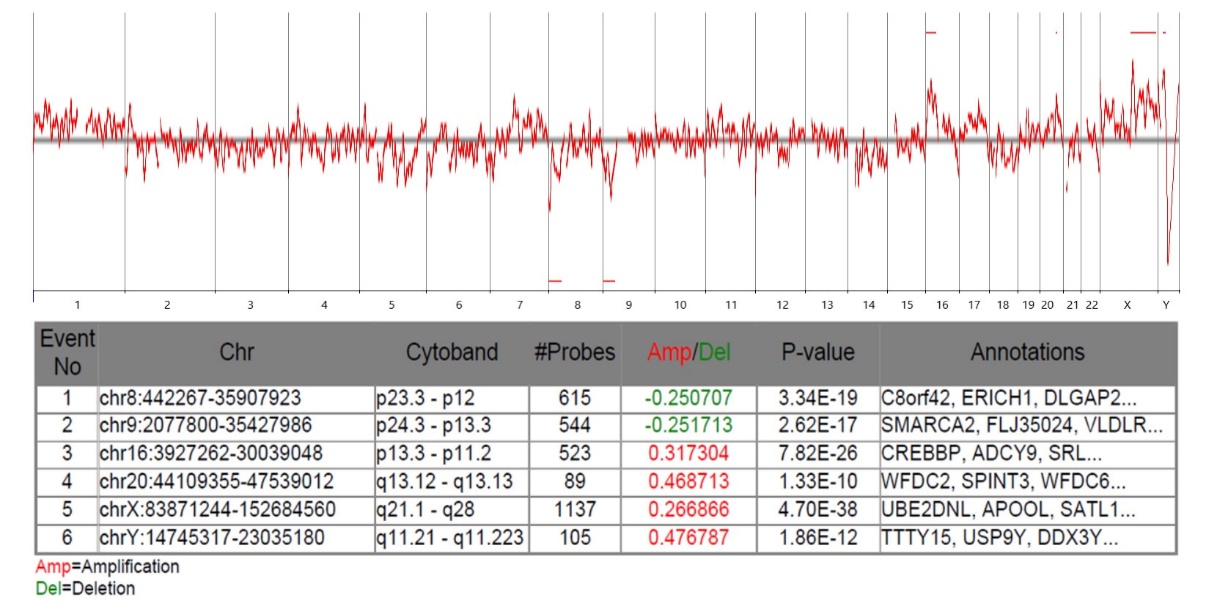


Case 13


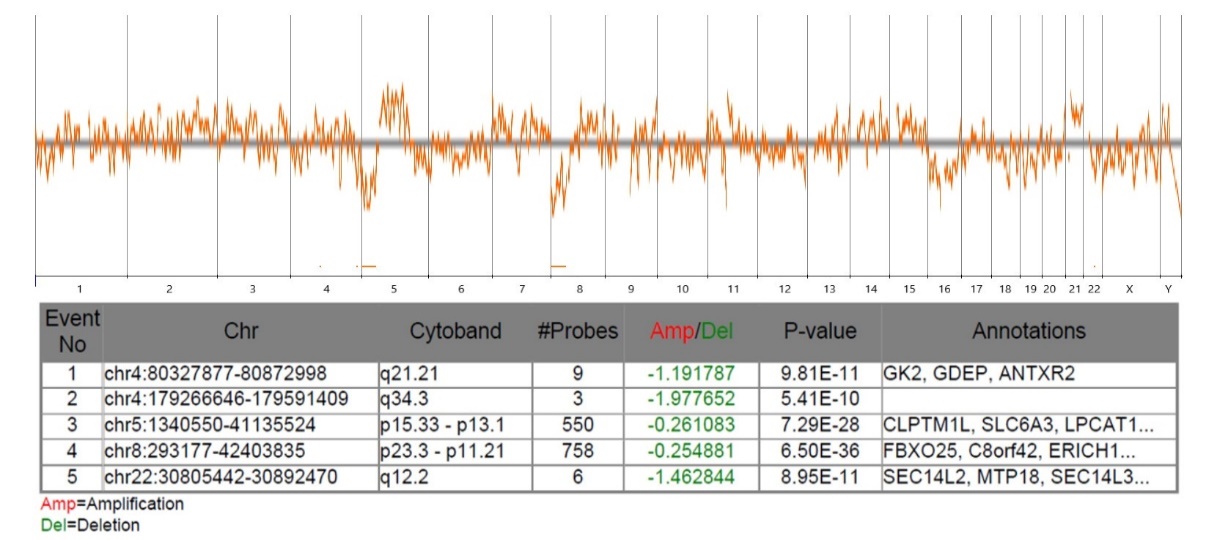


Case 14


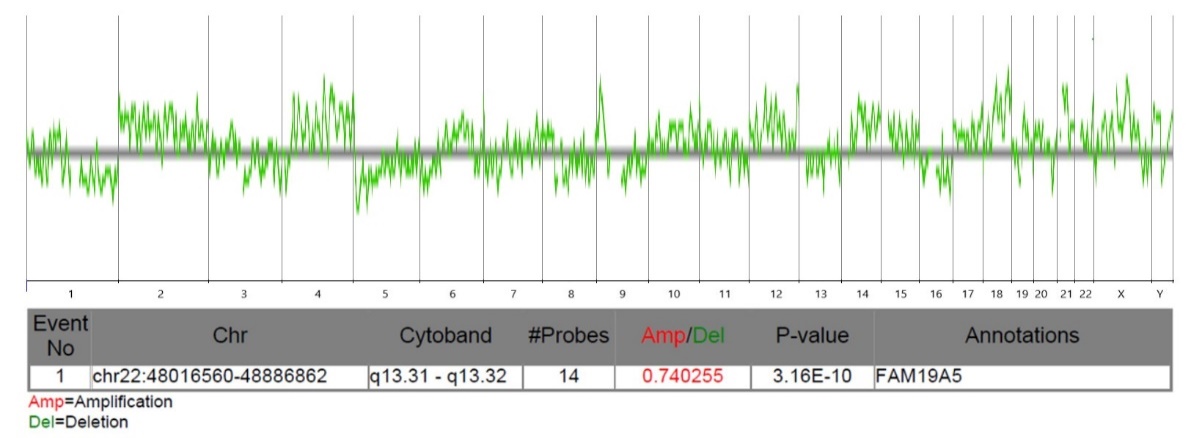


Case 15


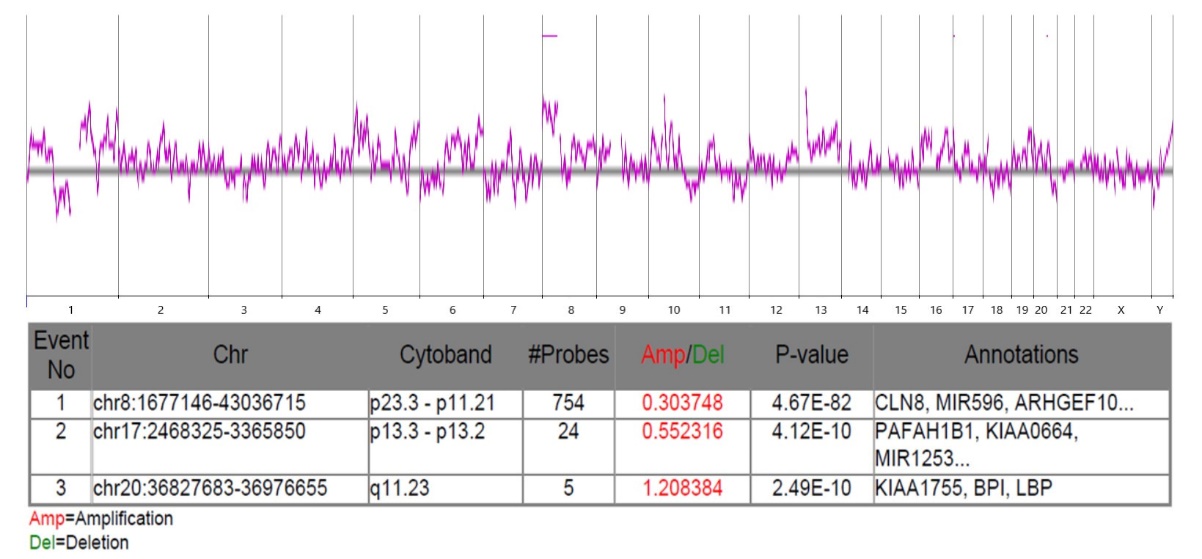


Case 16


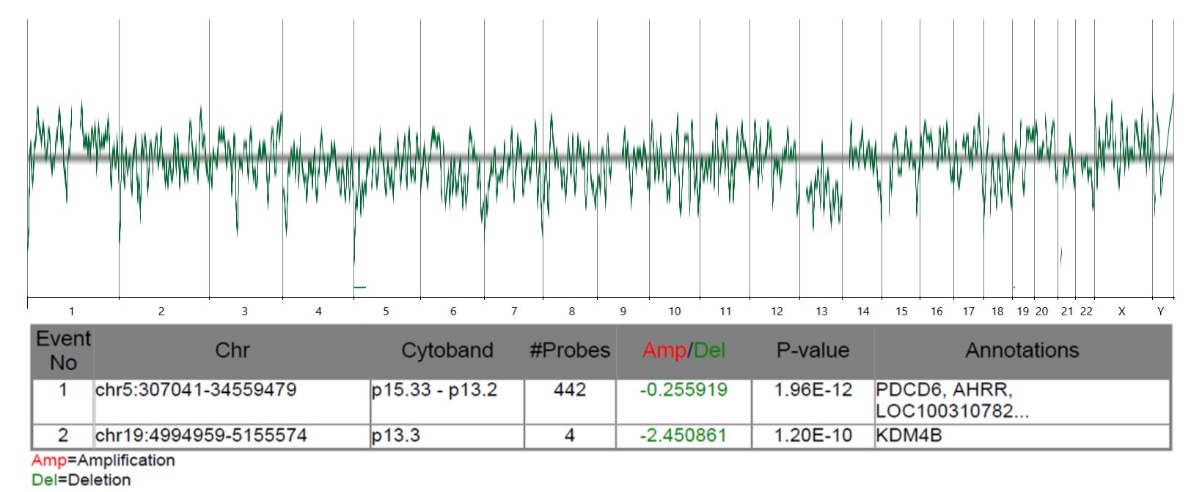


Case 17


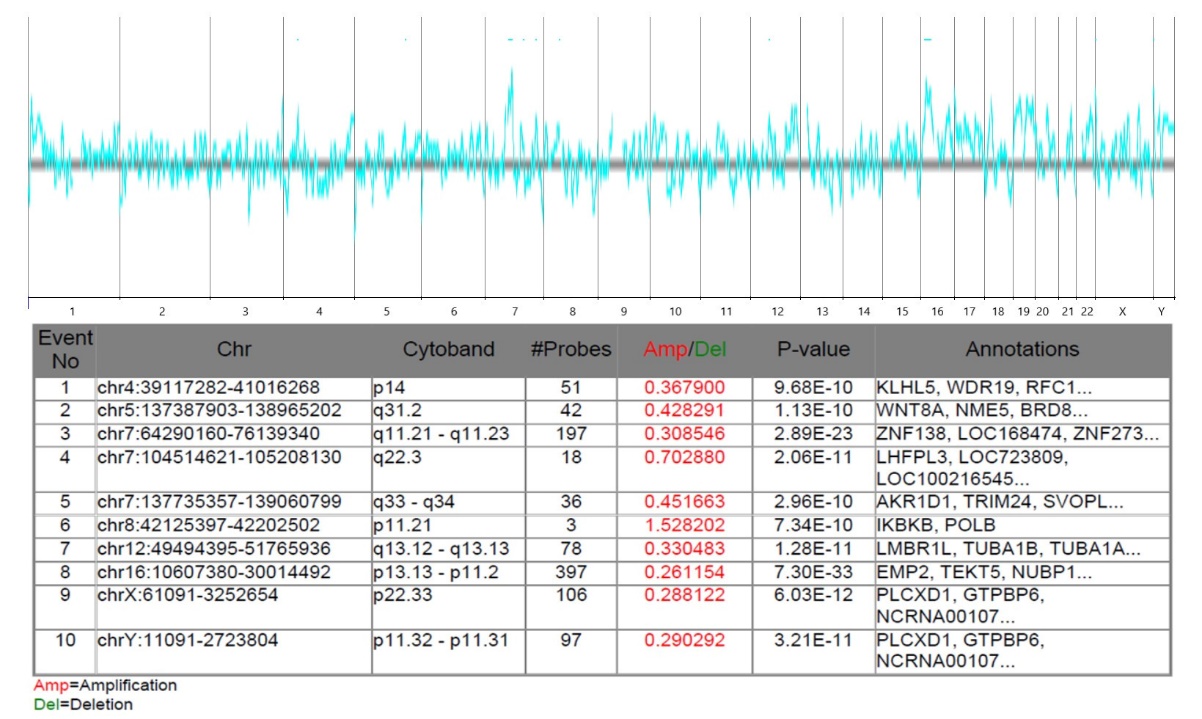


**Supplementary Fig. S3.** Molecular analysis by array comparative genomic hybridisation. All cases displayed amplification or deletion in one tumour, and the results suggested that these diagnoses were consistent with multiple primary lung cancer. Chr, chromosome; Amp, amplification; Del, deletion.


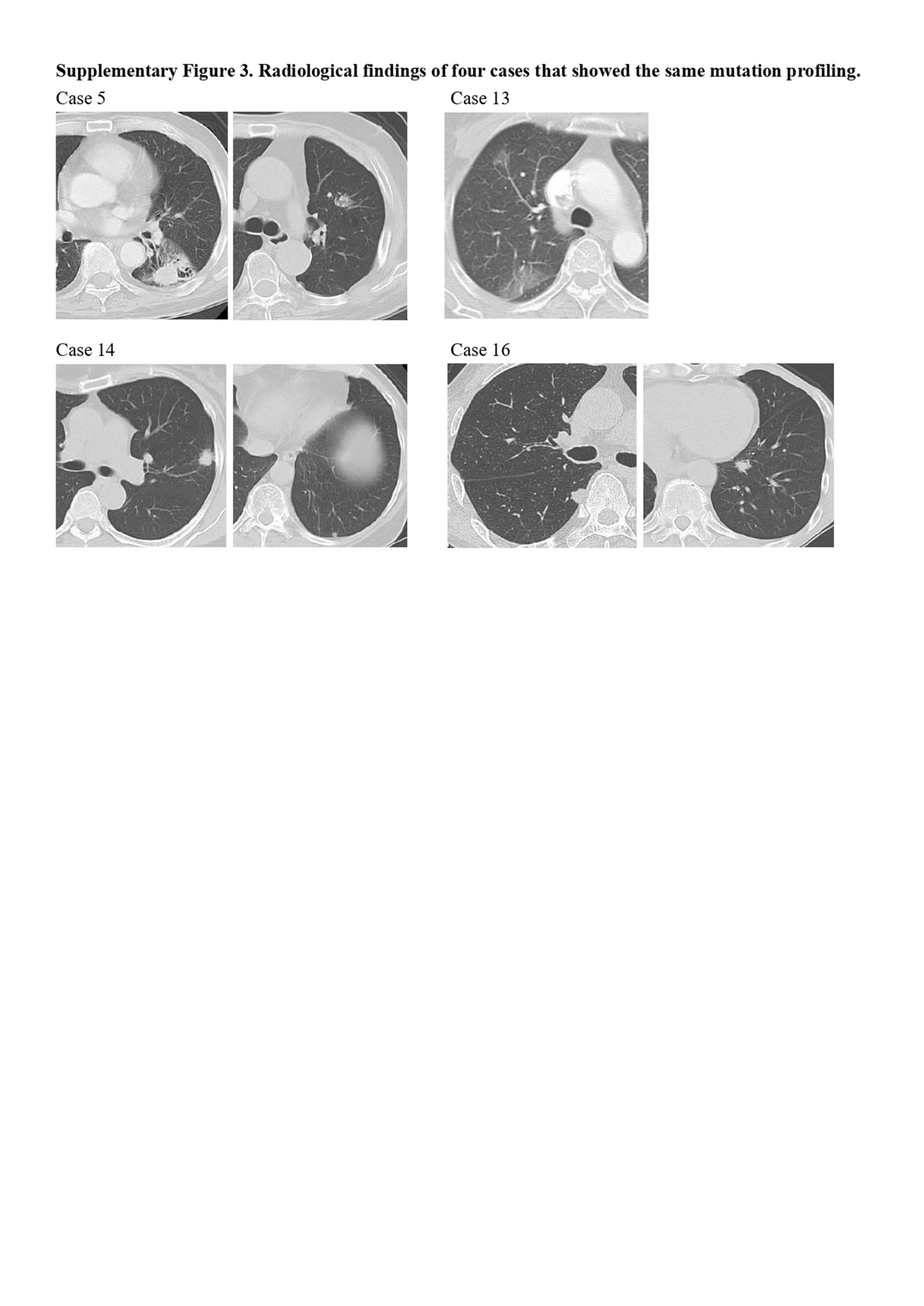


**Supplementary Fig. S4.** Radiological images of four cases with multiple lesions showing the same mutation profile. In Case 5, 13, and 14, multiple tumours were located in the same lobe but within different segments. Case 16 showed multiple tumours were located in the different lobes.

| Gene | ALL | Never/Light smoker | Middle/Heavy smoker | Male | Female | <70 | ≥70 |  |
| --- | --- | --- | --- | --- | --- | --- | --- | --- |
|  |  |  |  |  |  |  |  |  |
|  | % | % | % | % | % | % | % |  |
| [*EGFR*](http://www.genecards.org/cgi-bin/carddisp.pl?gene=CSMD3) | 41.0 | 59.3 | 16.6 | 21.3 | 58.0 | *25.0 | *45.5 |  |
| [*IDH1*](http://www.genecards.org/cgi-bin/carddisp.pl?gene=KDM5C) | 0.2 | 0.5 | 0.0 | 0.0 | 0.4 | *6.3 | *0.0 |  |
| [*KRAS*](http://www.genecards.org/cgi-bin/carddisp.pl?gene=KRAS) | 8.3 | 4.3 | 13.8 | 11.7 | 5.4 | *25.0 | *18.2 |  |
| [*NFE2L2*](http://www.genecards.org/cgi-bin/carddisp.pl?gene=NSD1) | 3.3 | 0.6 | 6.8 | 6.0 | 0.9 | *6.3 | *0.0 |  |
| [*PARP1*](http://www.genecards.org/cgi-bin/carddisp.pl?gene=PARP1) | *5.3 | *13.3 | *0.0 | *0.0 | *18.2 | *12.5 | *0.0 |  |
| [*PIK3CA*](http://www.genecards.org/cgi-bin/carddisp.pl?gene=PIK3CA) | 3.3 | 2.3 | 4.5 | 4.2 | 2.5 | *0.0 | *4.5 |  |
| [*PTPN11*](http://www.genecards.org/cgi-bin/carddisp.pl?gene=PKHD1) | *2.6 | *0.0 | *4.3 | *3.7 | *0.0 | *6.3 | *0.0 |  |
| [*STK11*](http://www.genecards.org/cgi-bin/carddisp.pl?gene=PTPN11) | 0.6 | 0.2 | 1.2 | 1.0 | 0.2 | *6.3 | *0.0 |  |
| [*TP53*](http://www.genecards.org/cgi-bin/carddisp.pl?gene=STK11) | 26.5 | 15.6 | 41.0 | 36.6 | 17.8 | 43.8 | *22.7 |  |
| *ARID1A* | *5.3 | *0.0 | *8.7 | *7.4 | *0.0 | *6.3 | *4.5 |  |
| *BRAF* | *2.6 | *6.7 | *0.0 | *0.0 | *9.1 | *0.0 | *4.5 |  |
| *FBXW7* | *2.6 | *0.0 | *4.3 | *3.7 | *0.0 | *6.3 | *0.0 |  |
| *FLT4* | *2.6 | *6.7 | *0.0 | *0.0 | *9.1 | *0.0 | *4.5 |  |
| *KAT6B* | *2.6 | *0.0 | *4.3 | *3.7 | *0.0 | *0.0 | *4.5 |  |
| *KMT2C* | *7.9 | *0.0 | *13.0 | *11.1 | *0.0 | *0.0 | *13.6 |  |
| *MAGI1* | *10.5 | *13.3 | *13.0 | *18.5 | *0.0 | *12.5 | *13.6 |  |
| *NF1* | *2.6 | *0.0 | *4.3 | *3.7 | *0.0 | *6.3 | *0.0 |  |
| *NOTCH4* | *2.6 | *0.0 | *8.7 | *7.4 | *0.0 | *12.5 | *0.0 |  |
| *NRAS* | *2.6 | *0.0 | *4.3 | *3.7 | *0.0 | *6.3 | *0.0 |  |
| *ROS1* | *2.6 | *0.0 | *4.3 | *3.7 | *0.0 | *6.3 | *0.0 |  |
| *SYNE1* | *2.6 | *6.7 | *0.0 | *0.0 | *9.1 | *0.0 | *4.5 |  |

**Supplementary Table S1.** Mutation frequency of single primary lung carcinoma we set based on JME study and this study data. The frequency with asterisk (*) was calculated based on our study data.

| Sample No. | Mapped Reads | On Target | Mean Depth | Uniformity |
| --- | --- | --- | --- | --- |
| 1A | 12,635,340 | 99.28% | 809.90 | 93.63% |
| 1B | 32,228,373 | 99.10% | 2061.00 | 95.43% |
| 1C | 13,042,904 | 99.19% | 837.00 | 95.61% |
| 2A | 12,099,141 | 98.63% | 756.50 | 93.49% |
| 2B | 18,657,523 | 99.42% | 1187.00 | 88.64% |
| 3A | 13,247,501 | 99.29% | 848.20 | 95.22% |
| 3B | 12,945,169 | 99.25% | 832.40 | 96.02% |
| 4A | 14,481,400 | 99.39% | 944.00 | 96.07% |
| 4B | 13,360,072 | 99.24% | 845.30 | 93.76% |
| 5A | 9,566,947 | 99.04% | 604.50 | 89.90% |
| 5B | 24,008,113 | 99.46% | 1535.00 | 92.77% |
| 6A | 10,823,712 | 99.32% | 695.00 | 95.09% |
| 6B | 11,929,569 | 99.22% | 774.00 | 96.15% |
| 7A | 18,876,669 | 90.92% | 981.00 | 40.75% |
| 7B | 13,223,756 | 99.46% | 846.20 | 93.85% |
| 8A | 21,394,922 | 99.33% | 1378.00 | 96.65% |
| 8B | 18,753,764 | 99.38% | 1218.00 | 95.96% |
| 8C | 30,688,310 | 99.43% | 1934.00 | 89.45% |
| 8D | 29,985,327 | 99.30% | 1933.00 | 96.34% |
| 9A | 13,431,867 | 99.39% | 871.10 | 95.55% |
| 9B | 13,390,913 | 99.46% | 868.50 | 95.49% |
| 10A | 12,711,439 | 99.45% | 828.80 | 95.54% |
| 10B | 20,220,038 | 99.04% | 1325.00 | 96.70% |
| 11A | 25,932,223 | 98.58% | 1635.00 | 94.39% |
| 11B | 8,637,777 | 99.53% | 562.00 | 95.38% |
| 12A | 11,831,110 | 99.22% | 755.70 | 94.99% |
| 12B | 16,977,928 | 99.26% | 1078.00 | 96.22% |
| 13A | 14,555,351 | 99.23% | 930.70 | 92.51% |
| 13B | 9,199,904 | 99.32% | 595.70 | 90.46% |
| 14A | 11,468,819 | 99.47% | 743.30 | 93.30% |
| 14B | 15,725,351 | 99.33% | 1001.00 | 95.12% |
| 15A | 10,315,435 | 99.15% | 664.70 | 95.24% |
| 15B | 9,446,433 | 99.12% | 606.30 | 96.11% |
| 16A | 11,385,242 | 99.03% | 721.60 | 95.29% |
| 16B | 11,520,892 | 99.40% | 733.90 | 95.98% |
| 17A | 27,982,950 | 98.94% | 1790.00 | 95.40% |
| 17B | 8,238,963 | 99.54% | 540.40 | 74.07% |

**Supplementary Table S2.** Results of targeted sequencing.

| Gene | Never/Light smoker (n=7) | | | *P* | Middle/Heavy smoker (n=10) | | | *P* | male (n=12) | | | *P* | female (n=5) | | | *P* | age < 70 (n=7) | | | *P* | age ≥ 70 (n=10) | | | *P* |
| --- | --- | --- | --- | --- | --- | --- | --- | --- | --- | --- | --- | --- | --- | --- | --- | --- | --- | --- | --- | --- | --- | --- | --- | --- |
|  | all | either | neither |  | all | either | neither |  | all | either | neither |  | all | either | neither |  | all | either | neither |  | all | either | neither |  |
| *EGFR* | 4 | 1 | 2 | .026 | 1 | 2 | 7 | .116 | 2 | 1 | 9 | .008 | 3 | 2 | 0 | .214 | 2 | 0 | 5 | .005 | 3 | 3 | 4 | .067 |
| *IDH1* | 0 | 0 | 7 | .938 | 0 | 1 | 9 | 1 | 0 | 1 | 11 | 1 | 0 | 0 | 5 | .956 | 0 | 1 | 6 | .751 | 0 | 0 | 10 | 1 |
| *KRAS* | 2 | 0 | 5 | <.001 | 0 | 3 | 7 | .761 | 2 | 2 | 8 | .007 | 0 | 1 | 4 | .911 | 1 | 1 | 5 | .008 | 1 | 2 | 7 | 0.115 |
| *NFE2L2* | 0 | 0 | 7 | .919 | 0 | 1 | 9 | .616 | 0 | 1 | 11 | .587 | 0 | 0 | 5 | .914 | 0 | 1 | 6 | .751 | 0 | 0 | 10 | 1 |
| *PARP1* | 1 | 0 | 6 | .023 | 0 | 0 | 10 | 1 | 0 | 0 | 12 | 1 | 1 | 0 | 4 | .036 | 1 | 0 | 6 | .023 | 0 | 0 | 10 | 1 |
| *PIK3CA* | 0 | 0 | 7 | .719 | 0 | 1 | 9 | .781 | 0 | 1 | 11 | .745 | 0 | 0 | 5 | .779 | 0 | 0 | 7 | 1 | 0 | 1 | 9 | .746 |
| *PTPN11* | 0 | 0 | 7 | 1 | 0 | 1 | 9 | .746 | 0 | 1 | 11 | .744 | 0 | 0 | 5 | 1 | 0 | 1 | 6 | .751 | 0 | 0 | 10 | 1 |
| *STK11* | 0 | 1 | 6 | 1 | 0 | 0 | 10 | .793 | 0 | 1 | 11 | .975 | 0 | 0 | 5 | .978 | 0 | 1 | 6 | .751 | 0 | 0 | 10 | 1 |
| *TP53* | 1 | 1 | 5 | .074 | 1 | 6 | 3 | .597 | 1 | 6 | 5 | .437 | 1 | 1 | 3 | .093 | 1 | 4 | 2 | .521 | 1 | 3 | 6 | .205 |
| *ARID1A* | 0 | 0 | 7 | 1 | 0 | 2 | 8 | .708 | 0 | 2 | 10 | .702 | 0 | 0 | 5 | 1 | 0 | 1 | 6 | .751 | 0 | 1 | 9 | .746 |
| *BRAF* | 0 | 1 | 6 | .751 | 0 | 0 | 10 | 1 | 0 | 0 | 12 | 1 | 0 | 1 | 4 | .758 | 0 | 0 | 7 | 1 | 0 | 1 | 9 | .746 |
| *FBXW7* | 0 | 0 | 7 | 1 | 0 | 1 | 9 | .746 | 0 | 1 | 11 | .744 | 0 | 0 | 5 | 1 | 0 | 1 | 6 | .751 | 0 | 0 | 10 | 1 |
| *FLT4* | 0 | 1 | 6 | .751 | 0 | 0 | 10 | 1 | 0 | 0 | 12 | 1 | 0 | 1 | 4 | .758 | 0 | 0 | 7 | 1 | 0 | 1 | 9 | .746 |
| *KAT6B* | 0 | 0 | 7 | 1 | 0 | 1 | 9 | .746 | 0 | 1 | 11 | .744 | 0 | 0 | 5 | 1 | 0 | 0 | 7 | 1 | 0 | 1 | 9 | .746 |
| *KMT2C* | 0 | 0 | 7 | 1 | 0 | 3 | 7 | .708 | 0 | 3 | 9 | .697 | 0 | 0 | 5 | 1 | 0 | 0 | 7 | 1 | 0 | 3 | 7 | .708 |
| *MAGI1* | 0 | 1 | 6 | .751 | 0 | 3 | 7 | .708 | 0 | 4 | 8 | .709 | 0 | 0 | 5 | 1 | 0 | 2 | 5 | .724 | 0 | 2 | 8 | .708 |
| *NF1* | 0 | 0 | 7 | 1 | 0 | 1 | 9 | .746 | 0 | 1 | 11 | .744 | 0 | 0 | 5 | 1 | 0 | 1 | 6 | .751 | 0 | 0 | 10 | 1 |
| *NOTCH4* | 0 | 0 | 7 | 1 | 0 | 1 | 9 | .746 | 0 | 1 | 11 | .744 | 0 | 0 | 5 | 1 | 0 | 1 | 6 | .751 | 0 | 0 | 10 | 1 |
| *NRAS* | 0 | 0 | 7 | 1 | 0 | 1 | 9 | .746 | 0 | 1 | 11 | .744 | 0 | 0 | 5 | 1 | 0 | 1 | 6 | .751 | 0 | 0 | 10 | 1 |
| *ROS1* | 0 | 0 | 7 | 1 | 0 | 1 | 9 | .746 | 0 | 1 | 11 | .744 | 0 | 0 | 5 | 1 | 0 | 1 | 6 | .751 | 0 | 0 | 10 | 1 |
| *SYNE1* | 0 | 1 | 6 | .751 | 0 | 0 | 10 | 1 | 0 | 0 | 12 | 1 | 0 | 1 | 4 | .758 | 0 | 0 | 7 | 1 | 0 | 1 | 9 | .746 |

**Supplementary Table S3.** Number of cases in each gene mutation for each non-intrinsic factors. Significant difference (*P* < .05) suggest the mutations would occur concomitantly, whereas no significant difference (*P* ≥ .05) suggest the mutations would occur by chance in the multiple tumors within the same individuals. all, mutations in all lesions within the same individuals.
